# Distribution, expression and methylation analysis of positively selected genes provides insights into the evolution in *Brassica rapa*

**DOI:** 10.1371/journal.pone.0256120

**Published:** 2021-10-08

**Authors:** Yue Guo, Jing Liu, Xingna Wang, Ying Li, Xilin Hou, Jianchang Du

**Affiliations:** 1 Provincial Key Laboratory of Agrobiology, Institute of Crop Germplasm and Biotechnology, Jiangsu Academy of Agricultural Sciences, Nanjing, China; 2 Key Laboratory of Biology and Genetic Improvement of Oil Crops, Ministry of Agriculture of People’s Republic of China, Oil Crops Research Institute, Chinese Academy of Agricultural Sciences, Wuhan, China; 3 Institute of Farm Product Processing, Jiangsu Academy of Agricultural Sciences, Nanjing, China; 4 State Key Laboratory of Crop Genetics and Germplasm Enhancement, Key Laboratory of Biology and Germplasm Enhancement of Horticultural Crops in East China, College of Horticulture, Nanjing Agricultural University, Nanjing, China; Huazhong University of Science and Technology, CHINA

## Abstract

It is believed that positive selection is one of the major evolutionary forces underlying organism phenotypic diversification. Nevertheless, the characteristics of positively selected genes (PSGs), have not been well investigated. In this study, we performed a genome-wide analysis of orthologous genes between *Brassica rapa* (*B*. *rapa*) and *Brassica oleracea* (*B*. *oleracea*), and identified 468 putative PSGs. Our data show that, (1) PSGs are enriched in plant hormone signal transduction pathway and the transcription factor family; (2) PSGs are significantly lower expressed than randomly selected non-PSGs; (3) PSGs with tissue specificity are significantly higher expressed in the callus and reproductive tissues (flower and silique) than in vegetable tissues (root, stem and leaf); (4) the proportion of PSGs is positively correlated with the number of retained triplication gene copies, but the expression level of PSGs decay with the increasing of triplication gene copies; (5) the CG and CHG methylation levels of PSGs are significantly higher in introns and UTRs than in the promoter and exon regions; (6) the percent of transposable element is in proportion to the methylation level, and DNA methylation (especially in the CG content) has the tendency to reduce the expression of PSGs. This study provides insights into the characteristics, evolution, function, expression and methylation of PSGs in *B*. *rapa*.

## Introduction

The adaptive evolution of genes and genomes determine the morphology, behavior, physiological adaptation, species divergence and evolutionary innovation [1]. Therefore, the detection of genes under positive selection is important to understand the molecular basis underlying organism adaptive evolution [2]. Such positively selected genes (PSGs) usually carry advantageous mutations, which are favorable for individuals to adapt environment, easily to survive and to get more offsprings. In many cases, such advantageous mutations are lost by chance, but some are very lucky to quickly spread in a population and eventually to be fixed. Currently, several approaches had been developed to detect PSGs, including comparative or phylogenetic methods and population genetic methods [3]. The former relies on the patterns of substitutions between species and the later primarily utilizes the patterns of intraspecific polymorphism [4,5]. By using these approaches, PSGs were found to be mainly enriched in sensory perception, tumor suppression, apoptosis, immunity and defense and spermatogenesis [6,7]. Meanwhile, strong evidence for positive selection had also been reported, such as genes involved in sensory perception, host-pathogen interactions, immunity, and reproduction [5,8].

By comparing the ratio of non-synonymous substitution to the synonymous substitution (Ka/Ks), one could be able to detect many candidate genes under different selective modes. For example, the ratio of Ka/Ks greater than one (potential PSGs) could be indicative of positive selection acting on. Nevertheless, the majority of genes in a genome, in fact, could be detected to have a ratio of Ka/Ks lower than one, providing the evidence that purifying selection (or negative selection) had occurred. Previous reports had indicated that there is a significant negative correlation between Ka/Ks and expression level [3,9,10]. Therefore, it is not surprising to see that most PSGs are expressed at lower levels than non-PSGs in human, chimpanzee, macaque, mouse, rat and dog genomes [3]. Based on the patterns of expression and the relationship between Ka/Ks and expression level, scientists had also detected several PSGs candidates [11,12].

DNA methylation usually refers to adding an extra methyl to the 5’-end of a nuclear acid, and it has been regarded as an important epigenetic modification in the regulation process of gene expression and transposon silencing [13]. In most plants, DNA methylation mainly occurs at three sequence contexts, including CG, CHG, and a symmetric CHH (where H is A, C, or T) sites of the cytosine (5mC) [13,14]. Nevertheless, the methylation levels vary greatly depending on the regions located in a genome. Generally, the methylation level of centromere region in the chromosome is obviously high, and the transposon enriched regions always possess a quite higher methylated status than the genetic region [15]. In contrast, the levels of gene methylation are usually low. The methylated modification of promoter region could inhibit the gene expression, but the functions of the methylation in exon region remain unclear [16]. It has also been shown that genes with body-methylation (DNA methylation in coding regions) are prone to be longer, more functionally important, and conserved between orthologs [17,18].

*B*. *rapa* is one of the important vegetable crops, and is cultivated worldwide. It is a member of the family *Brassicaceae* (a total of 338 genera and 3,709 species) [19], including the model plant *Arabidopsis thaliana* [20]. *B*. *rapa* belongs to three diploid *Brassica* species in the classical triangle of U [21], and undergone additional whole-genome triplication (WGT) which had occurred between 13 and 17 million years ago [22,23]. Due to the agricultural importance, the whole genome sequencing of three major *Brassica* species (*B*. *rapa*, *B*. *oleracea* and *B*. *napus*) have been completed [24–26].

Considering the origin, relationship and genomic information in *Brassicaceae* is clear now, we are wondering whether PSGs in *Brassica* have some specific features, such as functional importance, unique expression patterns, and different methylation levels. If so, they may play an important role in *Brassica* evolution and diversification. To do this, we first identified 468 PSGs candidates by comparing the ratio of Ka/Ks of orthologous genes between *B*. *rapa* and *B*. *oleracea*, and then further analyzed their distribution along chromosomes, their enrichment in pathways, expression level, and methylation properties. Our analysis provides evidence that PSGs have some unique characteristics, and may contribute to *Brassica* functional and phenotypic diversification. Our data also facilitate future utilization of PSGs and improvement of *Brassica* crop breeding.

## Materials and methods

### Sequence data

Gene and protein data sets for *B*. *rapa* and *B*. *oleracea* were downloaded from BRAD (http://brassicadb.cn/#/) [27]. Particularly, the coding sequences of *B*. *rapa* (/download_genome/data/rapa/Brassica_rapa.20100830.cds.tar) and *B*. *oleracea* (/download_genome/Brassica_Genome_data/BOL11/Scaffold.seq.110729_check.cds) was downloaded and extracted from the website (http://brassicadb.cn/#/Download/) for further analysis, respectively.

### Gene location in chromosome

We used MapInspect to map each positively selected gene to its corresponding chromosome location. The free soft was downloaded from the website (http://mapinspect.apponic.com/).

### Randomization analysis of the gene distribution

Randomization analysis of the genomic distribution of positively selected genes refers to previous studies [28].

### Orthologs and triplicates

The establishment of orthologs and sequence alignments were performed following the pipeline used in Yang et al [10]. Ks, Ka and their ratio Ka/Ks were estimated by yn00 module integrated in PAML package under default parameters [29]. Triplicates were identified based on the synthetic gene set between *A*. *thaliana* and three subgenomes of *B*. *rapa* and *B*. *oleracea*, respectively.

### Annotation and data analysis

#### GO annotation

The gene GO annotation data of *B*. *rapa* (V1.2) were downloaded from Phytozome (https://jgi.doe.gov/data-and-tools/phytozome/). Gene ontology and functional annotation was performed using the WEGO (http://wego.genomics.org.cn/cgi-bin/wego/index.pl). The GO enrichment analysis for the 468 PSGs, were performed by using hypergeometric tests and BLAST2GO with the 23817 *B*. *rapa*-*B*. *oleracea* orthologous gene pairs as the background. The GO term interaction was achieved using REVIGO (http://revigo.irb.hr/).

#### Pathway annotation

The KEGG pathway annotation was performed using the method reported [30], and the enrichment analysis was performed by using KOBAS2.0 [31,32]. The pathway display was accomplished using the online KEGG PATHWAY Database (http://www.kegg.jp/kegg/pathway.html).

#### Gene family classification

The resistance, transcription factors, flower, auxin and glucosinolate related genes were identified based on the classification of BRAD (http://brassicadb.cn/#/) [26,33]. The protein sequences of *A*. *thaliana* were used to identify the *B*. *rapa* homologs by BLASTP search with the lowest *E*-value as the best hit. Then the identification of protein kinase related genes were obtained based on the protein kinase superfamily classification in *A*. *thaliana* [34].

#### Expression pattern analysis

The whole genome transcriptomic data from six tissues (root, stem, leaf, flower, silique and callus) of Chiifu-401-42 were collected and were downloaded to estimate the expression level of each gene [35]. Venn Diagram and Heatmap for expression analysis were ploted by R3.0.3 (https://cran.r-project.org/bin/windows/base/old/3.0.3/).

#### DNA methylation analysis

The DNA methylation data from the whole genome of *B*. *rapa* were collected and were downloaded to estimate the methylation level of PSGs in this study [36].

## Results

### Non-random chromosomal distribution of PSGs in *B*. *rapa*

We first obtained 24219 orthologous gene pairs between *B*. *rapa* and *B*. *oleracea*, and then calculated the values of Ka, Ks and Ka/Ks [37]. After discarding 402 genes with extreme high Ks (Ks >0.3) [10], our data set contain 23817 orthologous pairs, including 468 strong potentially positively selective genes (Ka/Ks >1.2; [38]). We further mapped each PSG to the genome, and found that they were located unevenly in different chromosomes (S1 Fig and S1 Table). The densities of PSGs were also found to be associated with chromosomal locations according to both Chi-square test (*P* = 0.0179) and Fisher exact probability test (*P* = 0.0150) (S1 Table), indicating that genomic location is a factor influencing the distribution of PSGs.

We further divided PSGs into two groups, including PSGs with tissue specificity (expressed in only one tested tissue) and PSGs with constitutive expression (expressed in all six tested tissues). The data show that, relative to PSGs with constitutive expression, PSGs with tissue specificity are significantly non-randomly distributed, indicating that tissue specific PSGs are highly unevenly located along *B*. *rapa* chromosomes (Chi-square test and Fisher exact probability test, *P* < 0.05; S2–S4 Figs).

### Functional annotation and the enrichment of PSGs in *B*. *rapa*

**Fig 1:**
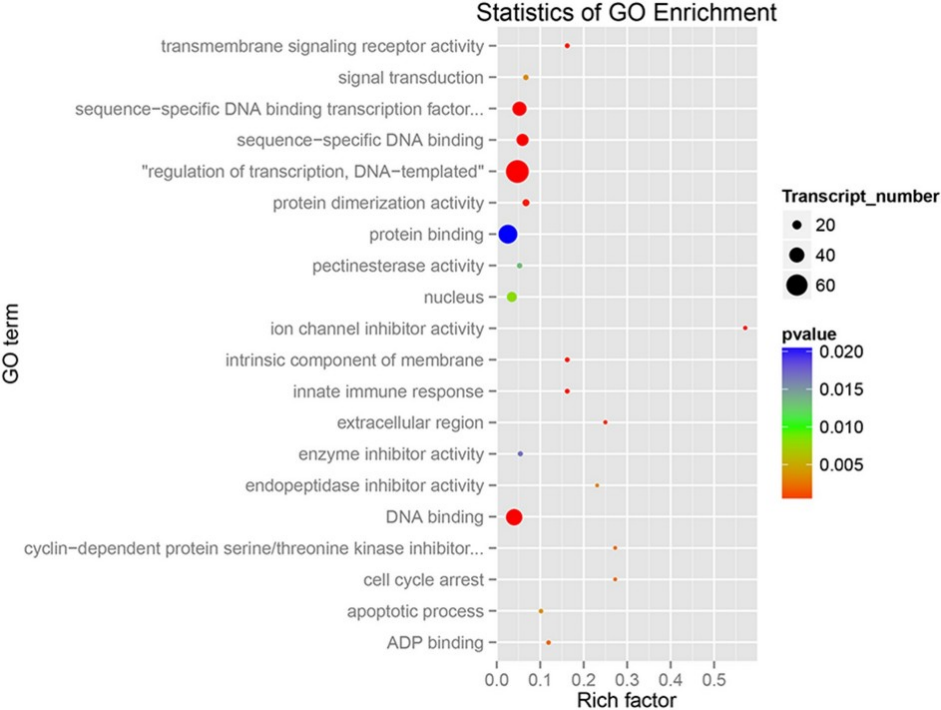
GO enrichment annotation. The different colors indicate the *p* values. Circle size suggests the number of significant GO term. Rich factor shows the ratio of positively selected genes to all orthologs in *B*. *rapa* genome.

In order to understand the gene function, we annotated PSGs in *B*. *rapa* by WEGO (S5 Fig), and then performed the enrichment analysis by Blast2GO (Fig 1; [39]). The interaction visualization was accomplished by REVIGO (http://revigo.irb.hr/), including three different GO categories, such as cellular component, molecular function and biological process (S6 Fig). The data show that PSGs are significantly enriched in a large number of functional categories, especially in the regulation of transcription belonging to biological process, DNA binding, and in the molecular function of sequence-specific DNA binding (*P* < 0.001; Figs 1 and S5).

**Fig 2:**
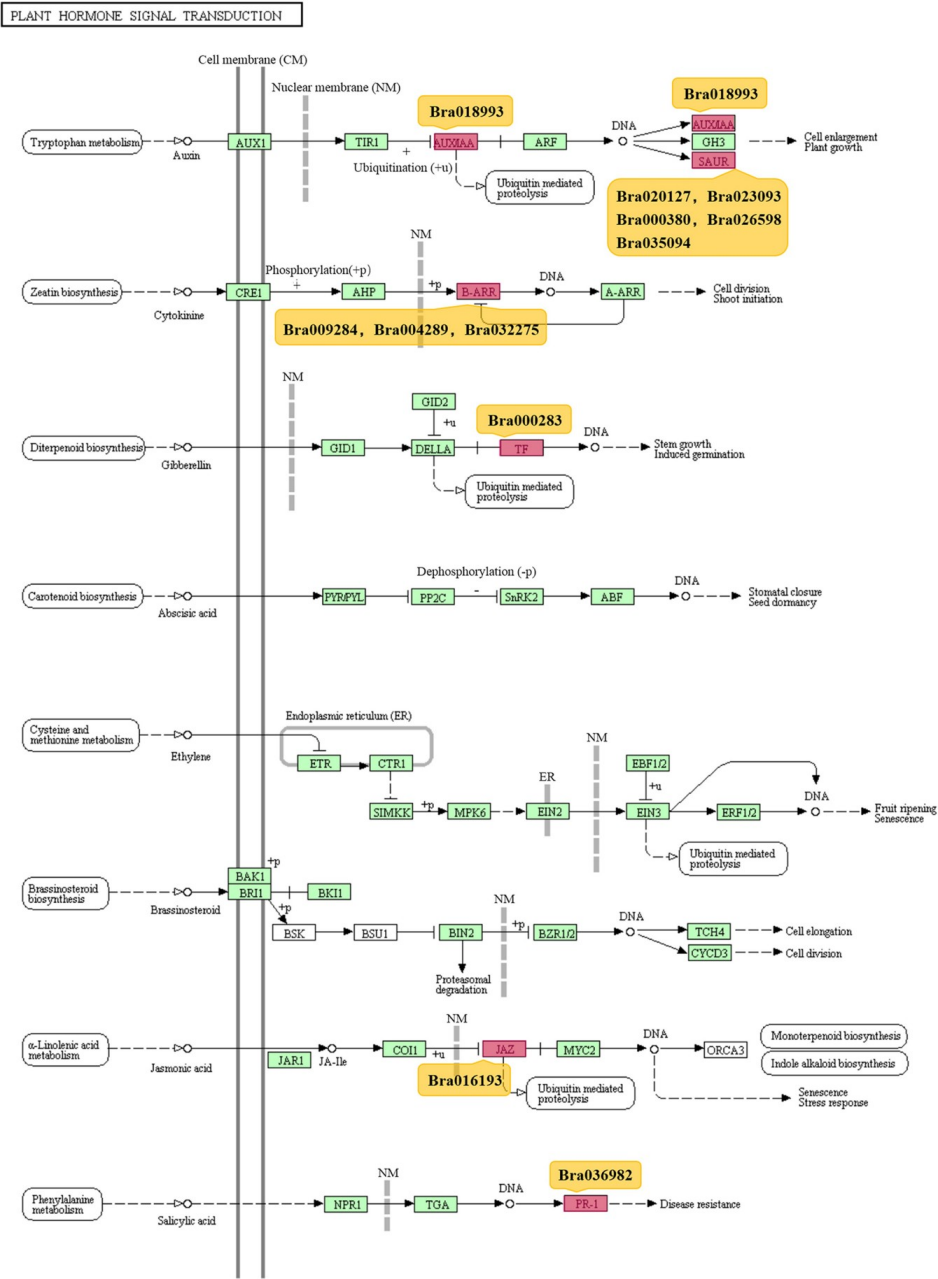
The display of enriched KEGG pathway of plant hormone transduction in positively selected genes.

Furthermore, we performed the KEGG pathway analysis for PSGs. Generally, PSGs were assigned into 29 KEGG pathways, belonging to 13 clades under five major KEGG categories, such as ‘Metabolism’, ‘Genetic information processing’, ‘Environmental information processing’, ‘Cellular processes’, and ‘Organismal systems’ (S7 Fig and S2 Table). In addition, 13 PSGs were found to be significantly enriched in the plant hormone signal transduction pathway, particularly in the process of tryptophan metabolism, zeatin biosynthesis, diterpenoid biosynthesis, *α*-linolenic acid metabolism, and phenylalanine metabolism (*P* < 0.001; Figs 2 and S7 and S2 Table).

To see whether PSGs are enriched in specific gene families, we classified PSGs into six families, such as *R* genes, protein kinase, transcription factors, flower genes, auxin genes and glucosinolate genes, according to previous reports [26,34]. The data show that 76 out of 89 PSGs (85.4%) belong to transcription factor family, particularly classified as MYB, NAC and HB types (Table 1 and S8 Fig). Nine auxin genes were also found to be PSGs and eight of them belong to the enriched plant hormone signal transduction pathway (Fig 2 and 1 and S2 Tables). *R* genes (2), protein kinase genes (1), and flower related genes (1) also contain PSGs, but no PSGs were detected in glucosinolate genes (Table 1). Both Chi-square test (*P* = 1.624e-05) and Fisher exact probability test (*P* = 5.00e-04) indicate that PSGs in *B*. *rapa* are significantly associated with gene types (Table 1).

**Table 1:** The number of positively selected genes and their proportion in the six gene families.

Group	R genes	Protein kinase	Transcription factors	Flower genes	Auxin genes	Glucosinolate genes
Positively selected gene	2	1	76	1	9	0
Non-positively selected gene	54	817	2301	95	199	65
Total number	56	818	2377	96	208	65

### The expression level and tissue specificity of PSGs in *B*. *rapa*

**Fig 3:**
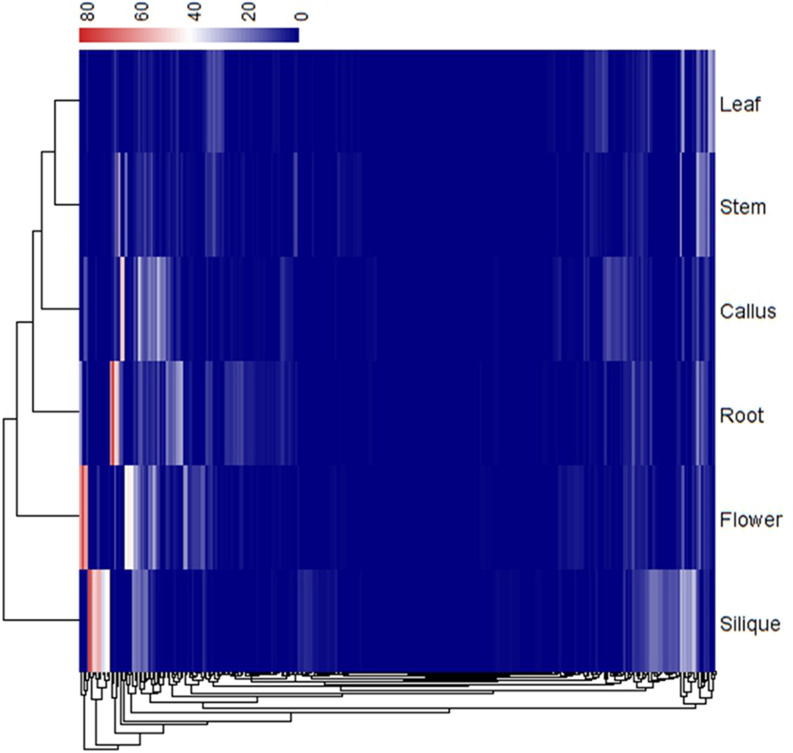
Cluster analysis of the positively selected genes for expression profile.

**Fig 4:**
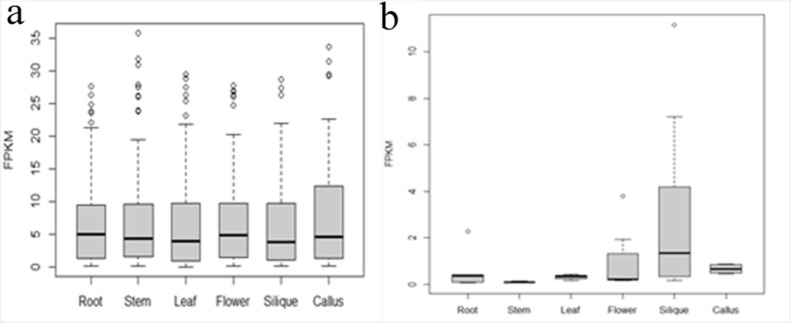
Comparisons of expression level of positively selected genes with constitutive expression (a) and positively selected genes with tissue specificity (b) in six *B*. *rapa* tissues (roots, stems, leaves, flowers, siliques and callus).

To understand the expression patterns of PSGs, we first investigated the gene expression level in *B*. *rapa*. As shown in Figs 3 and S9, overall PSGs are expressed significantly lower comparing to the randomly selected genes (Mann-Whitney U test, *P* < 1.50E-60). The tissues with the highest expression FPKM values for PSGs were found to be in the flower and root, and these genes are mainly located at Chr03, Chr05, Chr06 and Chr09 (Fig 4 and S3 Table). In contrast, the tissue with the lowest expression is in leaf, and PSGs are mainly located in Chr05 and Chr09 (Fig 3 and S3 Table). We also calculated the expression level of PSGs base on the chromosomes they belonging to, and found that PSGs in Chr03, Chr05, Chr06 and Chr09 had higher expression of FPKM values (S10 Fig), but the difference did not reach a significant level (Kruskal-Wallis rank sum test, *P* = 0.2868). The expression levels of different tissues in each chromosome were shown in detail (S11 Fig).

**Fig 5:**
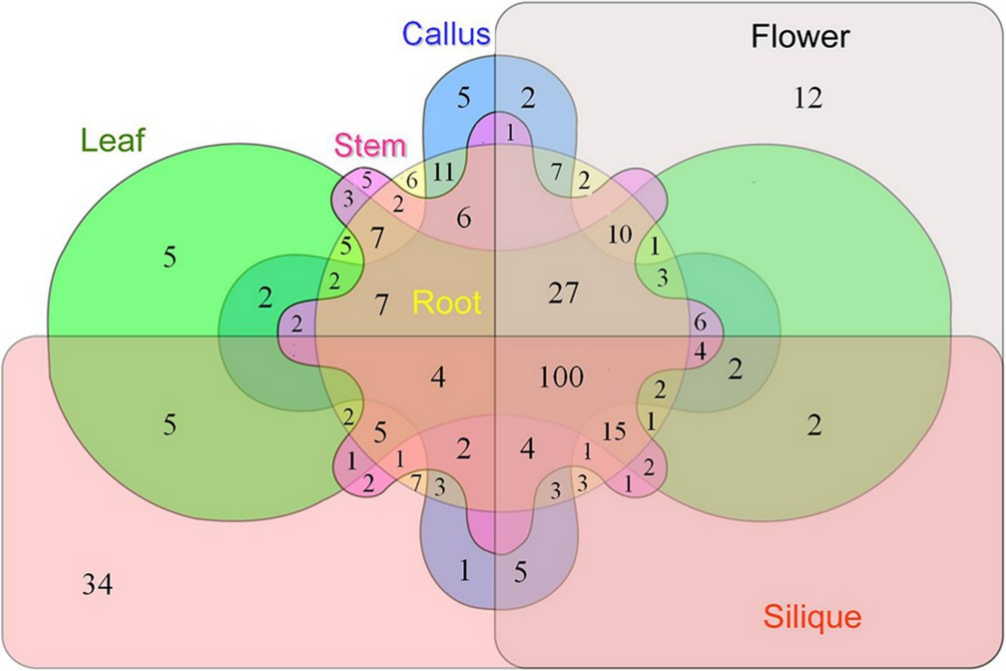
Venn diagram depicting the expression distribution of positively selected genes among six Chinese cabbage tissues, including roots, stems, leaves, flowers, siliques and callus.

There was also obvious difference was detected in expression level among tissue specific PSGs, and the highest FPKM value was found in silique (Kruskal-Wallis rank sum test, *P* = 0.002; Table 2 and Fig 4B). The numbers of the tissue specific PSGs in root, stem, leaf, flower, callus and silique are 6, 5, 5, 12, 5 and 34, respectively (Fig 5). In contrast, no significant difference of expression level was detected among the constitutive PSG groups (Kruskal-Wallis rank sum test, *P* = 0.7393; Table 2 and Fig 4A).

**Table 2:** The expression level in different tissues between constitutive and specific positively selected genes.

	Tissue constitutive	Tissue specificity
Root	7.0509 a	0.6394 a
Stem	7.4930 a	0.0909 a
Leaf	6.8945 a	0.3212 a
Flower	7.3077 a	0.9244 a
Silique	6.5893 a	2.5357 b
Callus	8.01294 a	0.6651 a

Data are the means±SD of expression level from every tissue. Different letters within columns indicate significant differences (*p* < 0.05) according to multiple comparison test after H-test.

The gene type (constitutive expression or specific expression) of PSGs was not associated with chromosomal distribution according to both Chi-square test (*P* = 0.3692) and Fisher exact probability test (*P* = 0.3843) (S3 Table). However, we found a significant difference between the number of tissue specific PSGs and the chromosome distribution, indicating that location is a factor influencing the density of PSGs with specific expression (KW test, *P* = 0.0011; S3 Table).

### The expression levels of PSGs appear to decay with the increase of WGT copies in *B*. *rapa*

**Fig 6:**
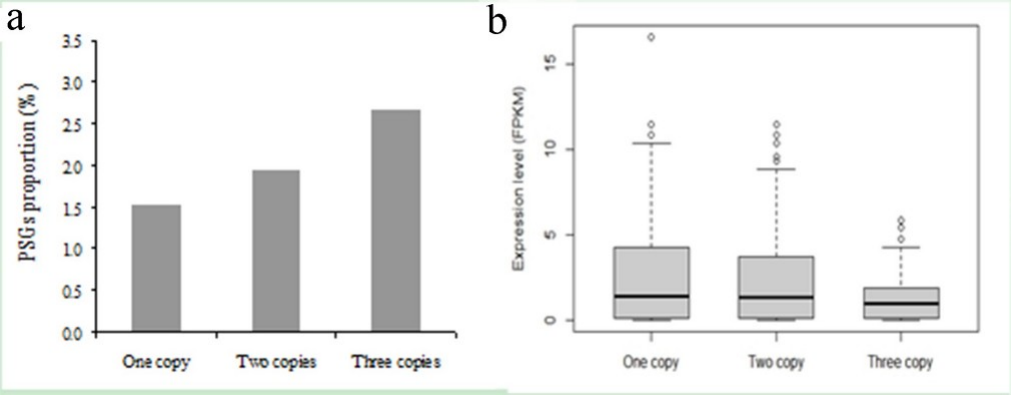
The proportion (a) and expression level (b) of positively selected genes for different type of triplicate genes, i.e, one, two or three copies retained after the triplication event. The band in the box is the median value, and the bottom and top of each box are the first (lower) and third (higher) quartiles. The ends of the whiskers indicate 1.5 interquartile range of first and third quartiles, respectively. Points outside the range are mild outliers.

The *B*. *rapa* genome had undergone a whole genome triplication (WGT) event after splitting with *Arabidopsis thaliana* from their common ancestor, following by a rediploidization process [26,36,40]. To understand the evolutionary fates and consequences of PSGs, we calculated the proportion of PSGs in each category (i.e. one copy, two copies, and three copies retained after the WGT event). Our data show that the percentage of PSGs increases with the number of WGT genes retained (from 1.53%, 1.94%, to 2.64%; Fisher exact probability test, *P* = 0.0665; Fig 6). Interestingly, the expression level of PSGs reduces with the increase of the number of WGT genes (from 2.921±0.5052, 2.677±0.4506, to 1.367±0.2265; H-test, *P =* 0.2434; Fig 6). Although both data sets do not reach the statistical tests, our data indicate that both the percentage of PSGs and the expression level of PSGs vary with the number of WGT genes retained.

### DNA methylation levels of PSGs vary among five genic regions in *B*. *rapa*

**Fig 7:**
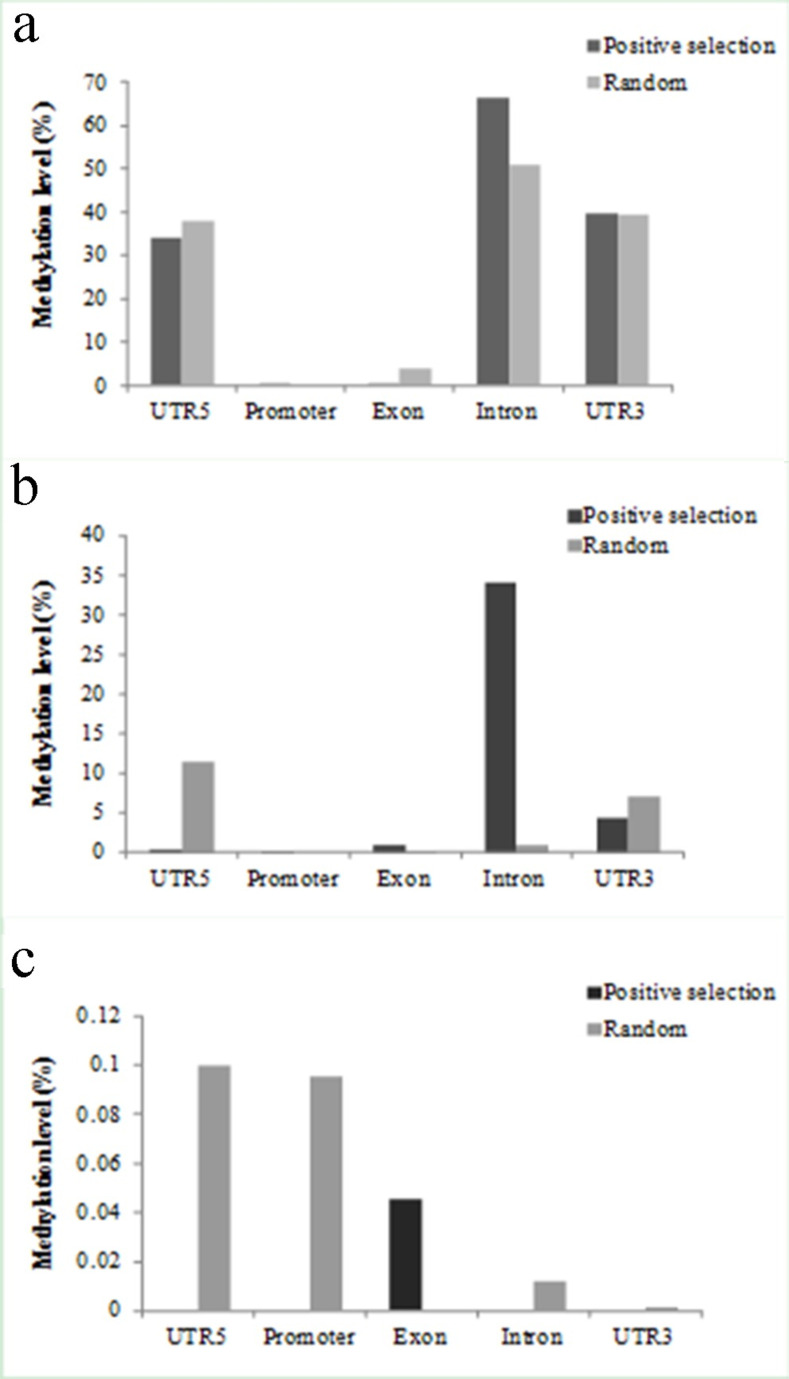
Comparisons of DNA methylation level between positively selected and randomly selected genes in different regions at CG (a), CHG (b) and CHH (c) contexts.

To understand the methylation levels of PSGs in different genic regions and at different methylation contexts in *B*. *rapa*, we divided a gene into five parts (UTR5, promoter, exon, intron and UTR3), and calculated the gene methylation levels using the data previously published [14]. Our data show that (1) the levels of PSG methylation vary depending on the genic regions and methylation contexts (*P* = 1.43e-03 for CG, *P* = 6.59e-04 for CHG, and *P* = 1.72e-04 for CHH context, according to the KW test; S4 Table); (2) high methylation (>30%) of PSGs were observed at CG context in UTR5, intron, and UTR3, and at CHG context in intron (Fig 7); (3) very low methylation (<0.1%) was detected at CHH context no matter for PSGs or for randomly selected genes (Fig 7); (4) for PSGs, significantly higher methylation was found at CHG context in intron (*P* = 3.20e-04) and significantly lower methylation was detected at CG in exon (*P* = 4.00e-04), at CHG in UTR5 (*P* = 9.719e-05), and at CHH context both in promoter (*P* = 3.49e-02) and in UTR5 (*P* = 9.00e-04), according to one-side Welch Two Sample *t*-test (Figs 7 and S12).

### Transposable element insertions contribute to gene methylation in *B*. *rapa*

As the major DNA components in the plant genomes, transposable elements (TEs) frequently insert into or close to functional genes, and thus can regulate genes epigenetically [13,41]. To understand if and to what extent TE insertions affect gene DNA methylation in *B*. *rapa*, we calculated the levels of gene methylation with and without insertions. At CG context with insertions, the gene methylation level increases obviously in promoter, exon, and UTR3 regions, but was not much affected in UTR5 and intron regions (H-test, *P* < 0.05); At CHG context with insertions, the gene methylation level increases quickly in UTR5, promoter, exon, and UTR3 regions, but decreases a lot in intron regions (H-test, *P* < 0.05); at CHH context with insertions, the gene methylation level increases in promoter, but reduces much in intron regions (H-test, *P* < 0.05; S13 Fig). At all three context CG, CHG, and CHH, the genes with insertions were all partially methylated, comparing to those genes without insertions, indicating that promoter may serve as a target for TE insertions and as an efficient way to regulate gene methylation (S13 Fig).

### Negative association of expression level with methylation level of PSGs in *B*. *rapa*

As we mentioned earlier, PSGs in reproductive organs (flower and silique, for example) are usually higher expressed than those in vegetative organs (root, stem and leaf) (Fig 5). To understand if such functional differences are associated with gene methylation, we compared the expression levels of PSGs with their methylation levels (S14 Fig). The data show that higher expression of PSGs are usually accompanied with lower methylation level, and a negative correlation was detected (Spearman Rank Correlation, *r* = -0.1070, *P* = 0.0333), indicating that methylation level may be associated with gene expression.

**Fig 8:**
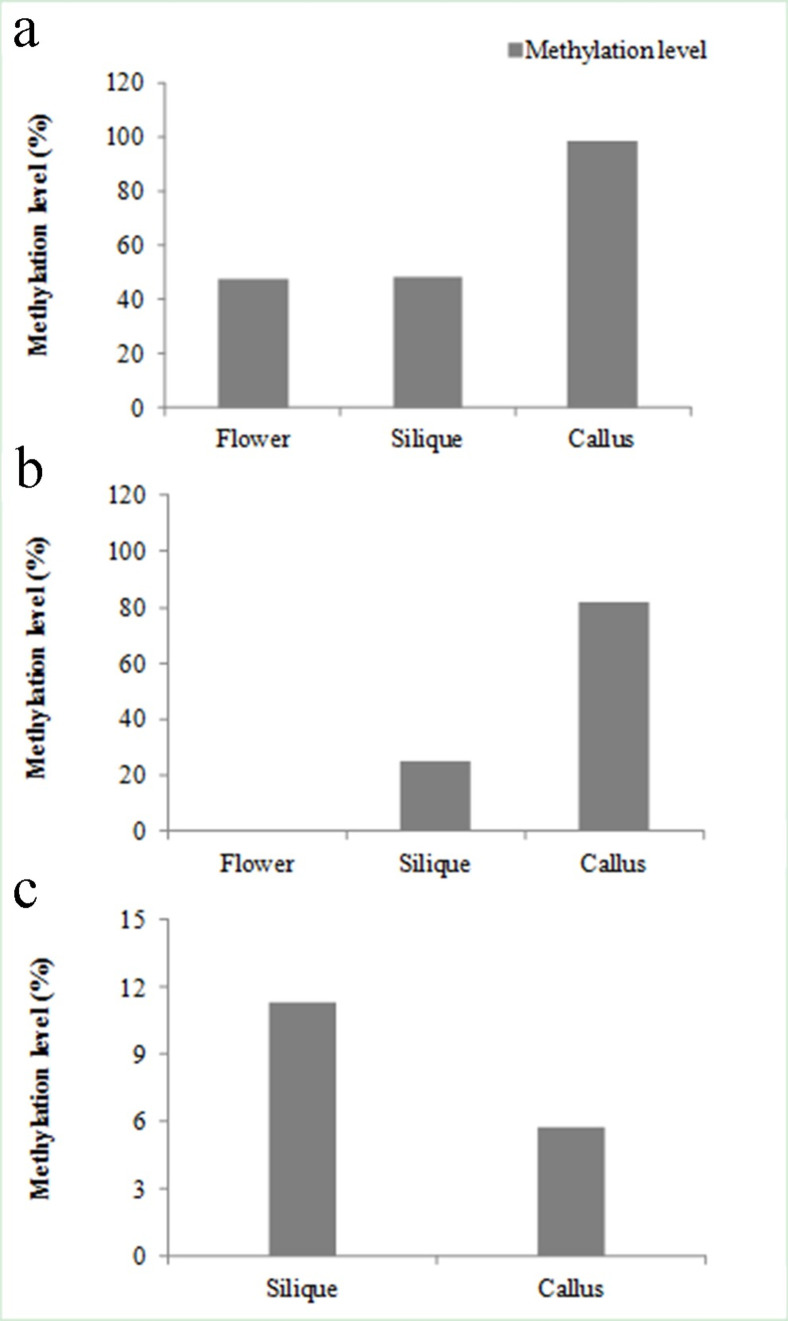
Comparisons of DNA methylation level of positively selected genes with tissue specificity in six *B*. *rapa* tissues (roots, stems, leaves, flowers, siliques and callus) at CG (a), CHG (b) and CHH (c) contexts.

Because some PSGs can only be expressed in one tissue (tissue specific), and some can express in every tissue (tissue constitutive), we are wondering if methylation levels are different between the two types of genes. Interestingly, for tissue specific PSGs, DNA methylation was only detected in flower, silique, and callus, and in these tissues, PSGs were highly methylated (Fig 8). For example, the methylation levels of PSGs had reached 40–100% at CG context, and 20–80% for CHG context. These genes were also been functionally annotated in details (S15 Fig). In contrast, for tissue constitutive PSGs, the methylation levels have little difference between different tissues, ranging from 13.98–23.47% at CG, 3.00–5.57% at CHG, and 6.59–7.24% at CHH contexts (S16 Fig).

## Discussion

### Features of PSGs in *B*. *rapa*: An overview

Although most protein coding genes in plants can be classifying into two categories: i.e., positively selected genes (PSGs) and negatively selected genes (NSGs), the characteristics of these two set of genes have not be comprehensively investigated. Our previous analysis of orthologous genes between *B*. *rapa* and *B*. *oleracea*, has revealed some structural, evolutionary, and expression features of PSGs [42]. For instance, PSGs usually have higher protein evolutionary rates, lower nucleotide protein evolutionary rates, shorter gene length, fewer exons, lower expression level, and higher tissue specific expression [42]. In this study, we further analyzed the properties of PSGs in *B*. *rapa*, including the chromosomal locations, biased distribution in different gene families, expression level and numbers of tissue specific expression in different tissues, correlations between WGT copies and expression levels, methylation levels in different genic regions, and the effects of insertions on gene expression levels. We also particularly investigated the difference between the two sets of PSGs in *B*. *rapa* (i.e., tissue specific PSGs and tissue constitutive PSGs), regarding their genomic distribution in different tissues, methylation levels at different contexts, and functional annotation. These analyses have revealed more comprehensive characteristics of PSGs in *B*. *rapa*. For example, PSGs are found to be biased distribution in different gene families, tissues, and genomic locations; PSGs expressed lower with the increase of WGT copies; PSGs are differentially methylated depending on different genic regions, methylation contexts and insertions (with or without); tissue specific PSGs and tissue constitutive PSGs differ in chromosomal locations, expression levels and methylation levels (Figs 3–8 and S1–S4 and S8–S16). These efforts not only establish a pine line to identify PSGs in plants, it is also helpful in revealing the whole picture of PSGs, and should be able to facilitate our understanding of how PSGs play an important role in the evolution and diversification process of *B*. *rapa*. The potential PSGs, as well as numerous favorable functional mutations revealed in this study, provide genetic targets for improvement and molecular breeding of *B*. *rapa*.

### The role of PSGs in hormone signal transduction pathway in *B*. *rapa*

Previous study has indicated that some particular genes, such as auxin-related genes, have been over-retained after WGT event, but the reasons and outcomes remain unclear [36]. In this study, we found that a total of 12 enriched PSGs had been involved in plant hormone signal transduction pathway (Fig 2). Half of these PSGs (Bra018993, Bra020127, Bra023093, Bra000380, Bra026598, Bra035094) participate in auxin related signal transduction pathway, and three PSGs (Bra009284, Bra004289, Bra032275) are involved in cytokinin related signal transduction (Fig 2). Because these two signal pathways are believed to be related with cell enlargement, plant growth, cell division and shoot initiation, the 13 enriched PSGs, therefore, may be partially responsible for the explanation of diversification during the fast evolution process of *Brassica* species [36].

The enrichment of PSGs were also been detected in other particular pathways. For example, the genes participating in the immunity and *p53* related pathways were found more to be PSGs in mammalian genomes [3]. These observations had been explained by that positive selection may frequently act directly on whole protein complexes or pathway [43,44]. Another hypothesis is that adaptive changes in one protein may have a “cascade effect”, leading to changes in other genes [43–45]. Although the PSG enrichment pathways are different between plants and mammalians, it seems clear now that positive selection play an important role in biological response and phenotypic formation.

### PSGs with specific expression is an indicator for expression abundance, expressed tissues and methylation level in *B*. *rapa*

Several studies had showed that significant negative correlations between Ka/Ks and expression level (abundance) were observed in *Arabidopsis* [10], yeast [9], and six mammalian genomes [3]. Our earlier analysis also indicated that PSGs (which is defined as higher Ka/Ks) tend to be lower expressed, comparing to NSGs [42]. These observations had been frequently found, but the underlying mechanisms are still debated. However, at least three hypothesis had been proposed, such as gene pleiotropy leading to selection increase [46], protein misfolding opposed by selection [47] and selection for translational efficiency [9]. We are not clear yet which hypothesis is truly responsible for the low expression level of PSGs. Perhaps two or more mechanisms need to be combined together, to explain why PSGs usually have low expression level.

In this study, we also show that tissue specific PSGs can serve as an indicator for expression abundance, as well as methylation level. For instance, the expression levels of PSGs with specific expression are significantly lower than those PSGs with constitutive expression (*P* << 0.0001, student *t*-test; Table 2). The former are highly methylated, but they were only be detected in flower, silique, and callus; the latter are lowly methylated, but they were detected across all six tested tissues (Figs 8 and S14).

### DNA methylation profiles of PSGs in *B*. *rapa*

The epigenetic modification of DNA is known as cytosine methylation, which is always associated with nucleosome positioning and histone modification. CG、CHG and CHH (where H is A, C, or T) are three sequence contexts of the cytosine (5mC) [13,14]. In this study, we investigated the gene methylation profiles in *B*. *rapa*. Our data show that (1) CG context is the major DNA methylation (Figs 7 and S14); (2) The methylation levels in the regions of intron and UTR are higher than those in the promoter and exon region at the CG and CHG context of PSGs (Fig 7A and 7B); (3) PSGs were methylated mainly in reproductive organs (flower and silique), and callus (Fig 8); (4) the methylation level was associated with TE insertions and expression level (S13 and S14 Figs). These observations are basically consistent with previous studies, indicating that PSGs participate in DNA methylation process in *B*. *rapa* [15,48–52]. It should be mentioned that transposable elements may not be fully annotated due to the difficulty for their assembly in the genome, and some could be missed. We are not clear how much it could affect the gene methylation analysis with or without transposable elements. In the meanwhile, the methylation levels could vary with environmental factors, biotic and abiotic stresses. Therefore, the methylation levels can only reflect the status at that particular condition, and relative intensity among different sequence contexts, and different genic regions.

It is particularly interesting to find that the promoter regions without insertions are free of methylation, and those regions with insertions have high levels of methylation (S13 Fig), providing the evidence that TEs mainly regulate gene expression by targeting gene promoters.

## Conclusions

We have initially identified 468 positively selected gene (PSGs) candidates by comparing the orthologous genes between two *Brassica* species, *B*. *rapa* and *B*. *oleracea*, and then further analyzed their distribution along chromosomes, the enrichment in pathways, expression patterns, and methylation properties. Our data support that PSGs are biased distributed depending on the families, tissues, and genomic locations; the methylation levels of PSGs vary in different genic regions, methylation contexts and status of insertions; tissue specific PSGs and tissue constitutive PSGs differ in chromosomal locations, expression levels and methylation levels. Our analysis provides evidence that PSGs have some unique properties, and may contribute to *Brassica* functional and phenotypic diversification. Our data may also facilitate gene functional study and future utilization of PSGs in *Brassica* crop breeding.

## Supporting information

S1 Fig Chromosomal location of PSGs in the *B*. *rapa* genome (Mbp). (TIF)

S2 Fig Chromosomal location of PSGs with tissue specificity in different tissues, including root (r), leaf (l), stem (st), flower (f), silique (si), and callus (c). (TIF)

S3 Fig Distribution of PSGs along 10 chromosomes in *B*. *rapa*. (TIF)

S4 Fig Randomization analysis of the genomic distribution of gene number between tissue specific or constitutive PSGs. (TIF)

S5 Fig GO classification of the PSGs in *B*. *rapa*. The 468 PSGs can be classifiable into three main categories as follows: Biological process, cellular component, and molecular function. In some cases, one PSG may have multiple terms.
(TIF)

S6 Fig The interactive graph view of GO terms in three main categories, including biological process (a), cellular component (b) and molecular function (c).
(TIF)

S7 Fig The number of the PSGs in each clade of the KEGG pathway. The PSGs were assigned into 29 KEGG pathways within 13 clades under five major categories: Metabolism (I), genetic information processing (II), environmental information processing (III), cellular processes (IV), and organismal systems (V).
(TIF)

S8 Fig The number of PSGs in different transcription factor families. (TIF)

S9 Fig Cluster analysis of expression profile for the random non-PSGs. (TIF)

S10 Fig The expression level of PSGs in the 10 chromosomes. The band in the box is the median value, and the bottom and top of each box indicates the first (lower) and third (higher) quartiles. The ends of the whiskers indicate 1.5 interquartile range of first and third quartiles, respectively. Points outside the range are mild outliers.
(TIF)

S11 Fig The expression level of the PSGs in 6 different tissues and in 10 different chromosomes. (TIF)

S12 Fig The p values of a paired T-test between PSGs and random non-PSGs by the heat map at CG (a), CHG (b) and CHH (c) contexts in B. rapa.
(TIF)

S13 Fig The methylation level of genes inserted by transposable elements (a) or not (b).
(TIF)

S14 Fig The methylation (a) and expression (b) level of the PSGs in six tissues at three different methylation contexts.
(TIF)

S15 Fig The KEGG pathway annotation of PSGs with tissue specificity in *B*. *rapa*, including metabolism (I), genetic information processing (II), environmental information processing (III). (TIF)

S16 Fig Comparisons of DNA methylation level of the PSGs with constitutive expression in six B. rapa tissues (roots, stems, leaves, flowers, siliques and callus) at CG (a), CHG (b) and CHH (c) contexts.
(TIF)

S1 Table The number of PSGs and their proportion in the ten chromosomes. (XLSX)

S2 Table The list of enriched KEGG genes and auxin-related genes. (XLSX)

S3 Table The number of expressed PSGs in different tissues and chromosomes. (XLSX)

S4 Table The *p* values of PSGs and random non-PSGs in five different genomic regions analyzed by Kruskal-Wallis rank sum test. (XLSX)

## Abbreviations

PSGs: Positively selected genes
WGT: Whole genome triplication
TEs: Transposable elements
UTR: Untranslated regions
Ka: The number of nonsynonymous substitutions per non-synonymous site
Ks: The number of synonymous substitutions per synonymous site
