# Supplementary figures and images for: Distribution, expression and methylation analysis of positively selected genes provides insights into the evolution in Brassica rapa
Source: PLoS One. 2021 Oct 8;16(10):e0256120. doi: 10.1371/journal.pone.0256120 (PMC8500406; doi:10.1371/journal.pone.0256120)

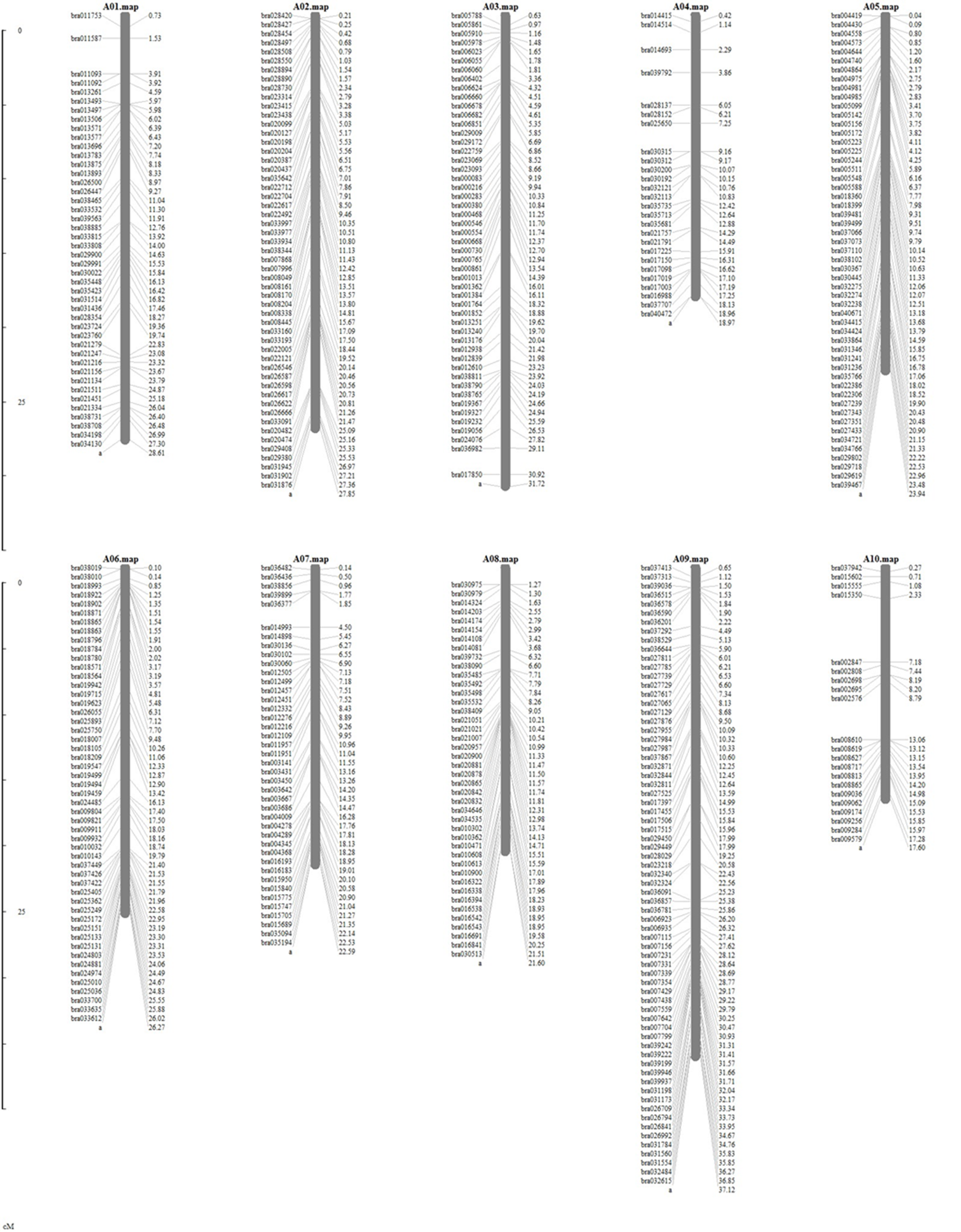

Supplement: S1 Fig — (TIF) [file pone.0256120.s001.tif]

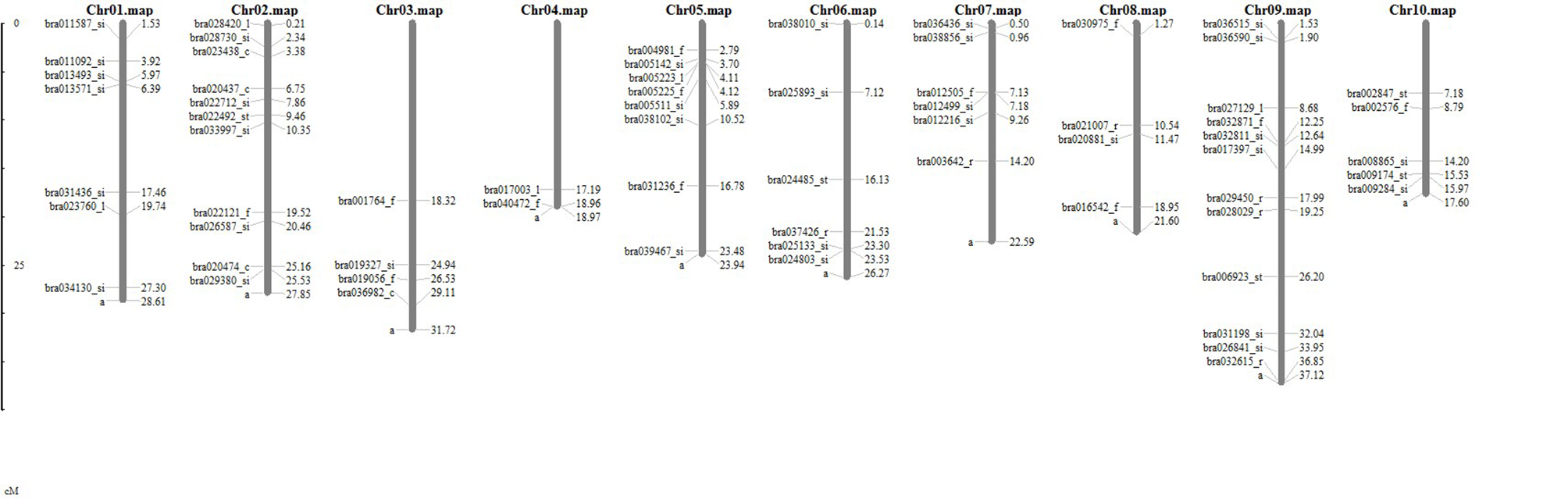

Supplement: S2 Fig — (TIF) [file pone.0256120.s002.tif]

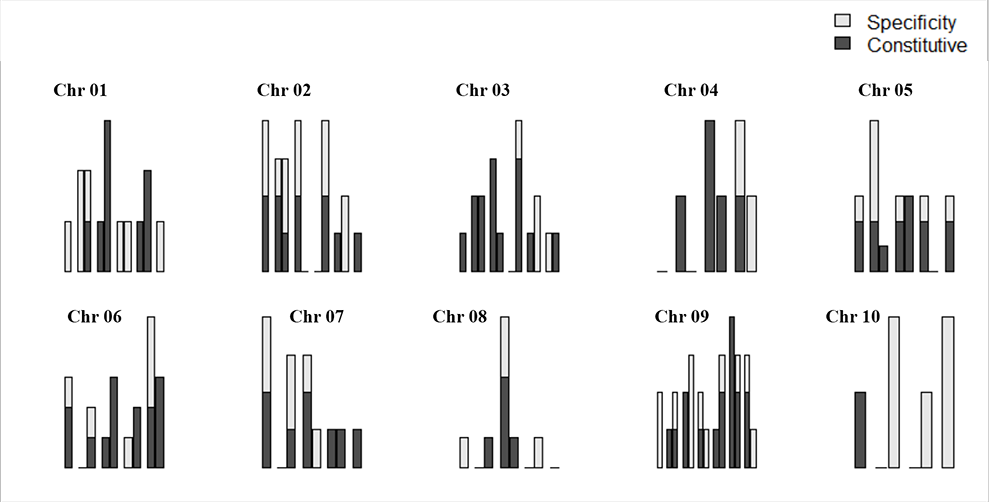

Supplement: S3 Fig — (TIF) [file pone.0256120.s003.tif]

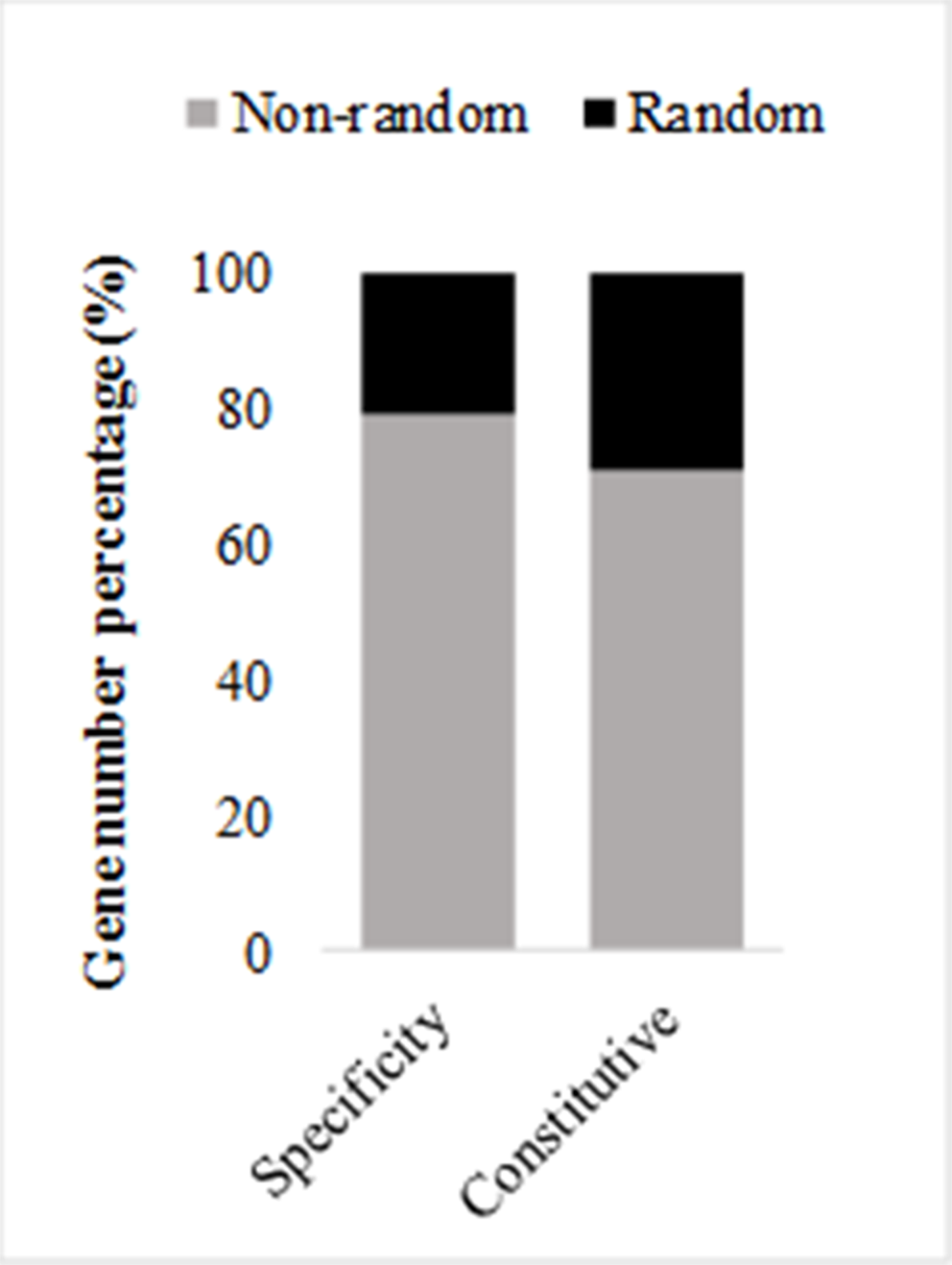

Supplement: S4 Fig — (TIF) [file pone.0256120.s004.tif]

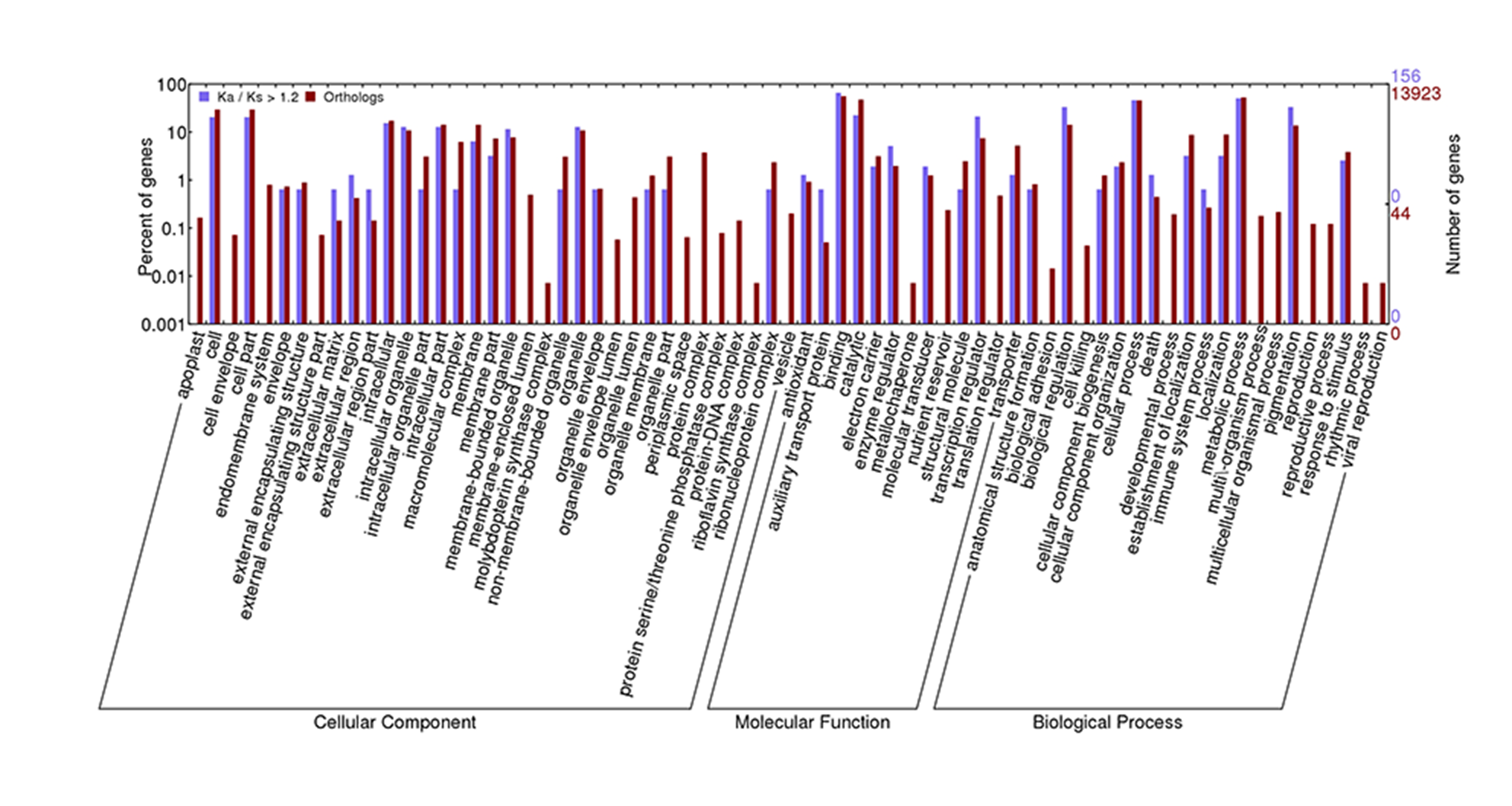

Supplement: S5 Fig — The 468 PSGs can be classifiable into three main categories as follows: Biological process, cellular component, and molecular function. In some cases, one PSG may have multiple terms. (TIF) [file pone.0256120.s005.tif]

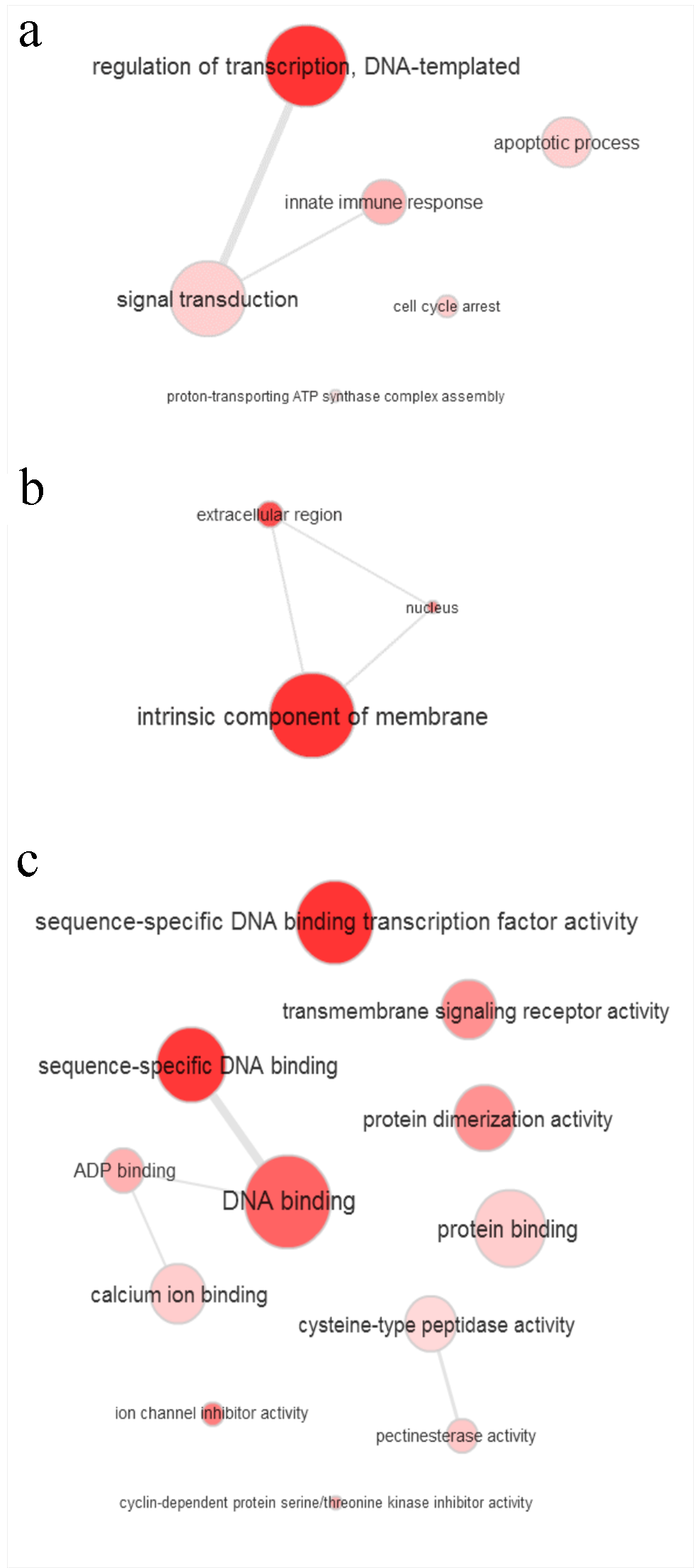

Supplement: S6 Fig — The interactive graph view of GO terms in three main categories, including biological process (a), cellular component (b) and molecular function (c). (TIF) [file pone.0256120.s006.tif]

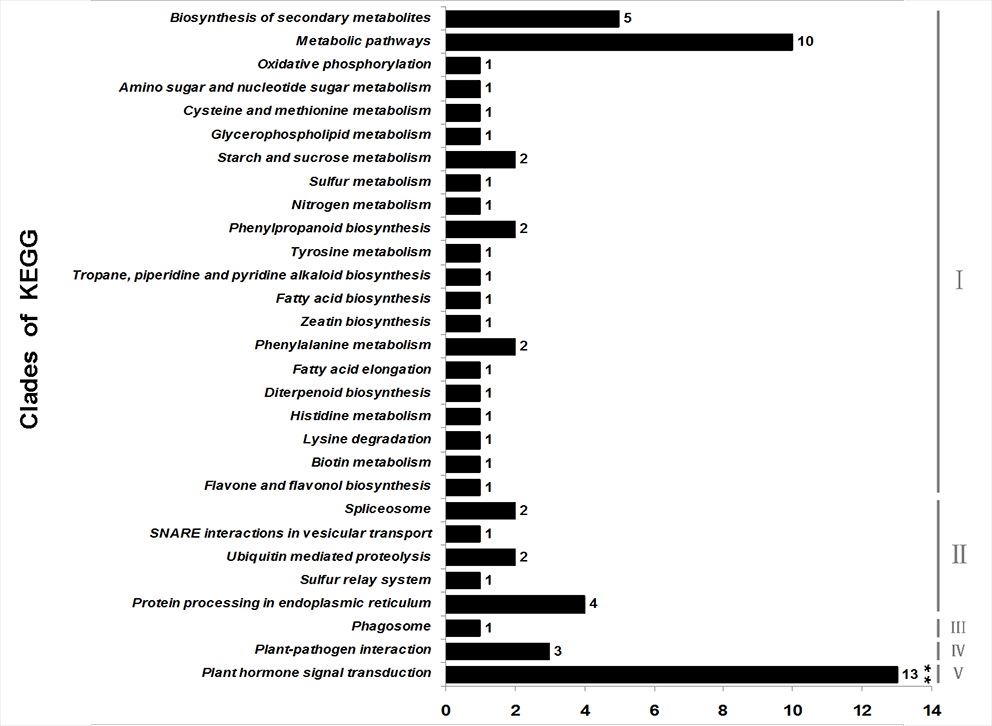

Supplement: S7 Fig — The PSGs were assigned into 29 KEGG pathways within 13 clades under five major categories: Metabolism (I), genetic information processing (II), environmental information processing (III), cellular processes (IV), and organismal systems (V). (TIF) [file pone.0256120.s007.tif]

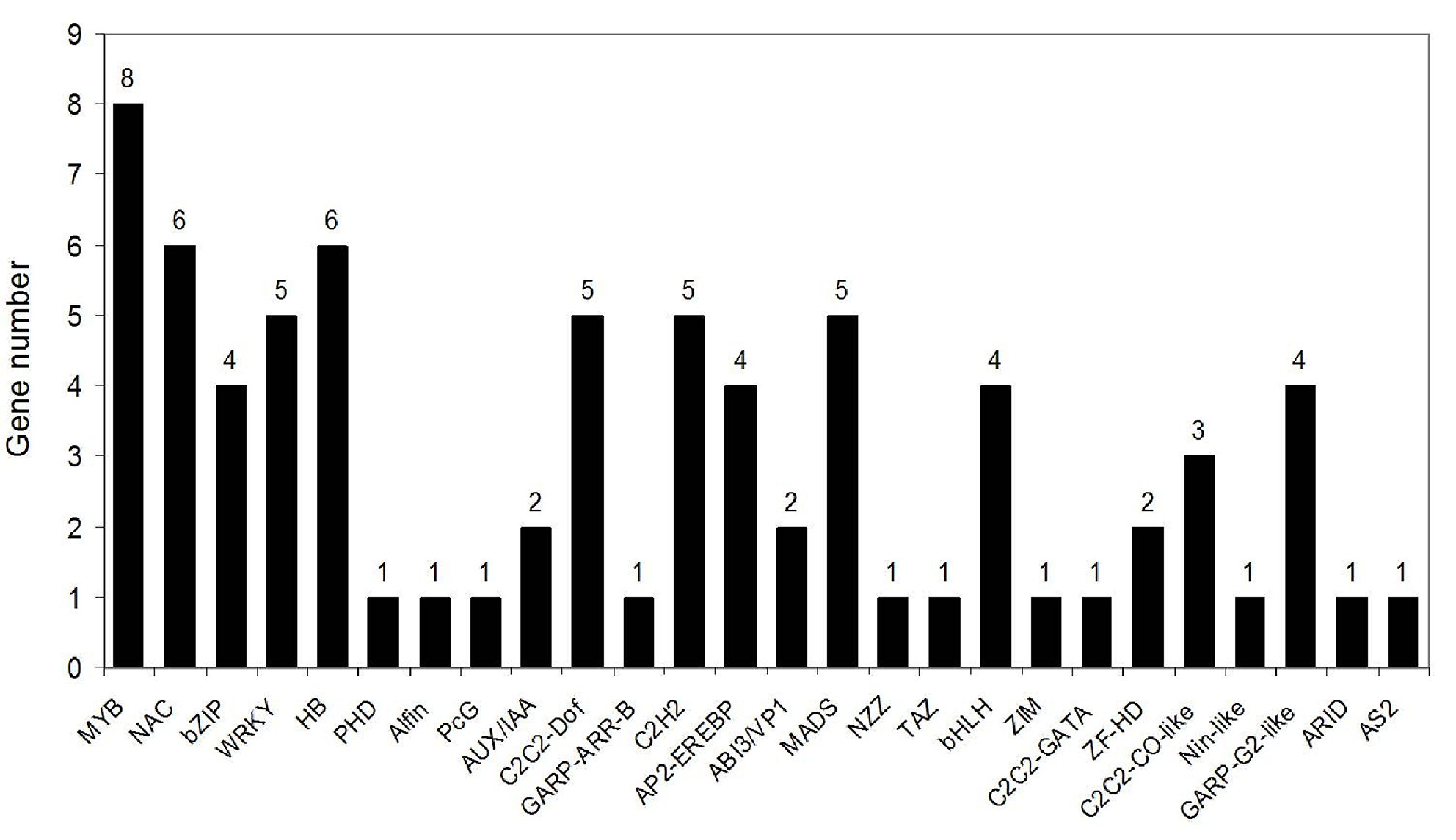

Supplement: S8 Fig — (TIF) [file pone.0256120.s008.tif]

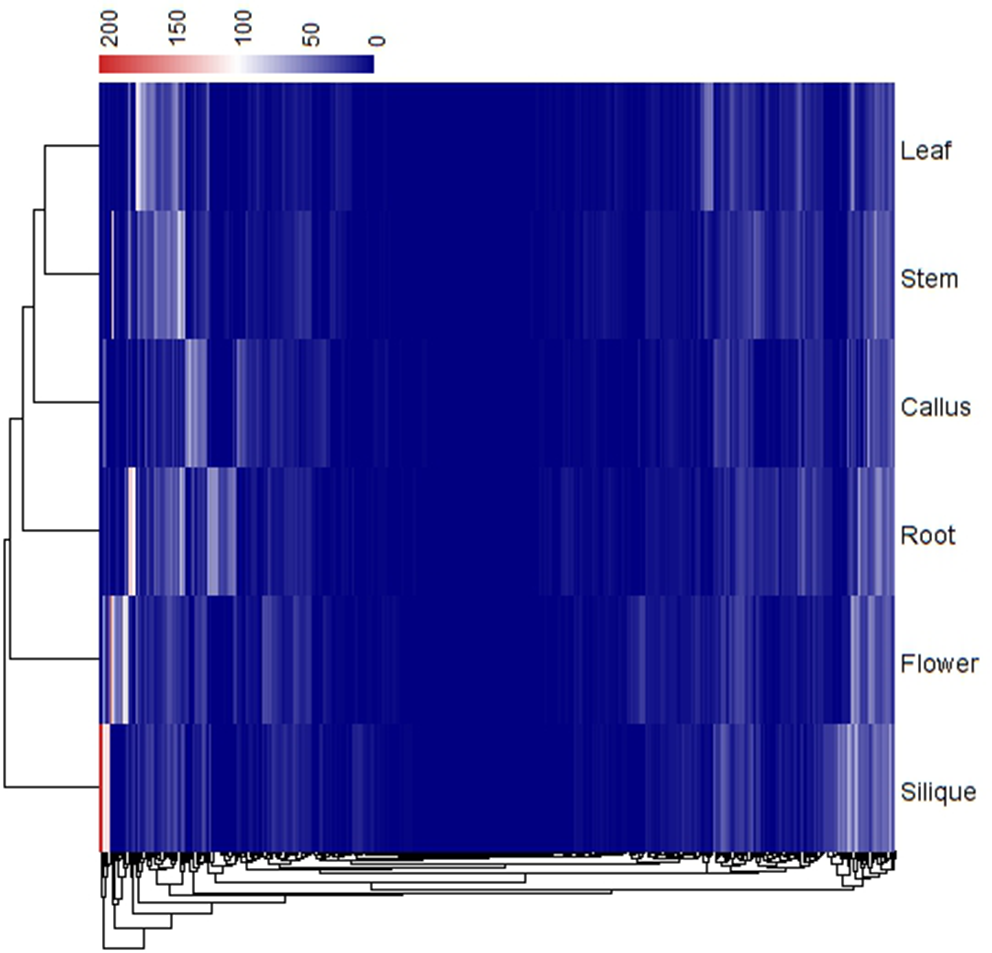

Supplement: S9 Fig — (TIF) [file pone.0256120.s009.tif]

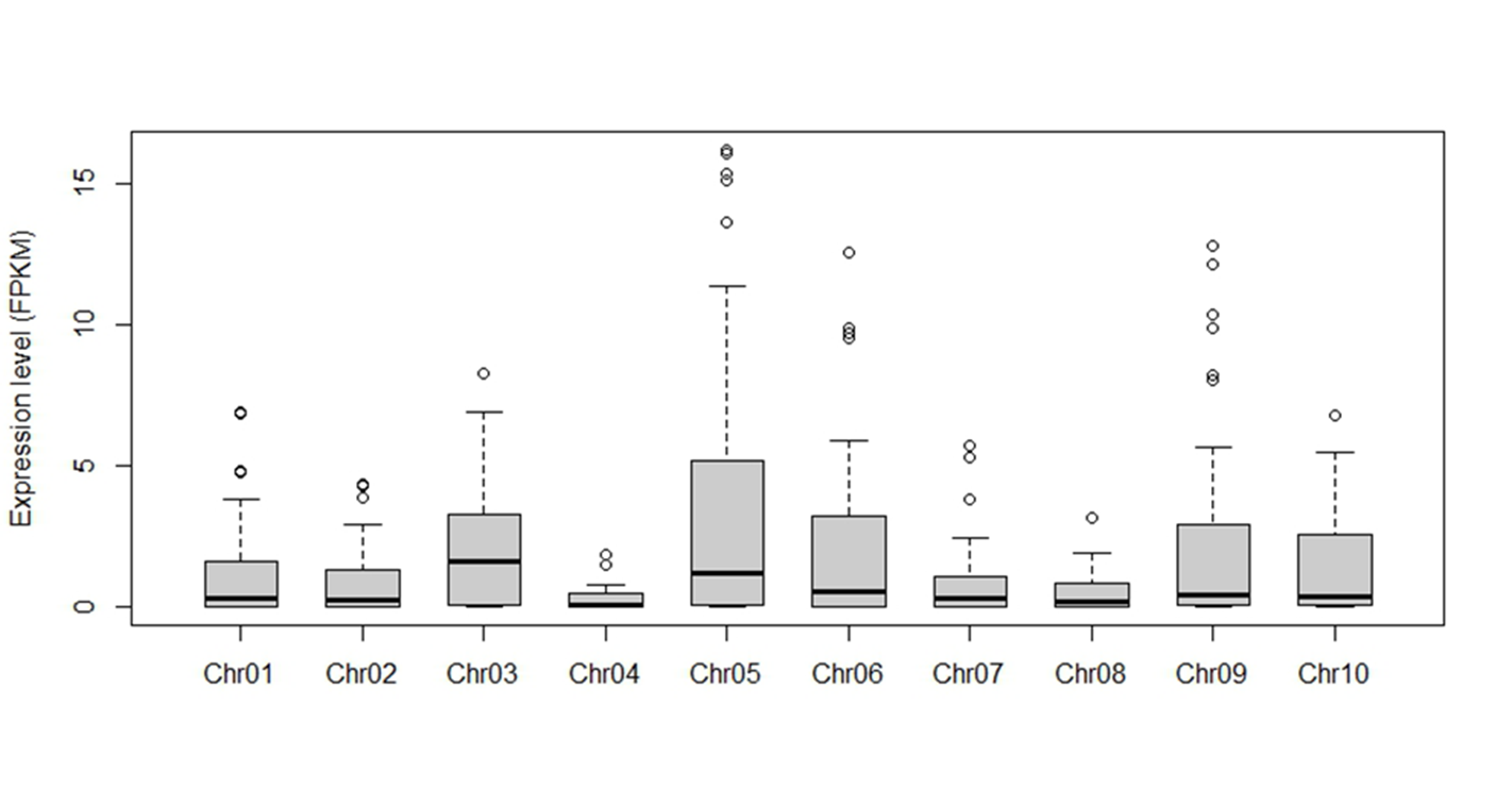

Supplement: S10 Fig — The band in the box is the median value, and the bottom and top of each box indicates the first (lower) and third (higher) quartiles. The ends of the whiskers indicate 1.5 interquartile range of first and third quartiles, respectively. Points outside the range are mild outliers. (TIF) [file pone.0256120.s010.tif]

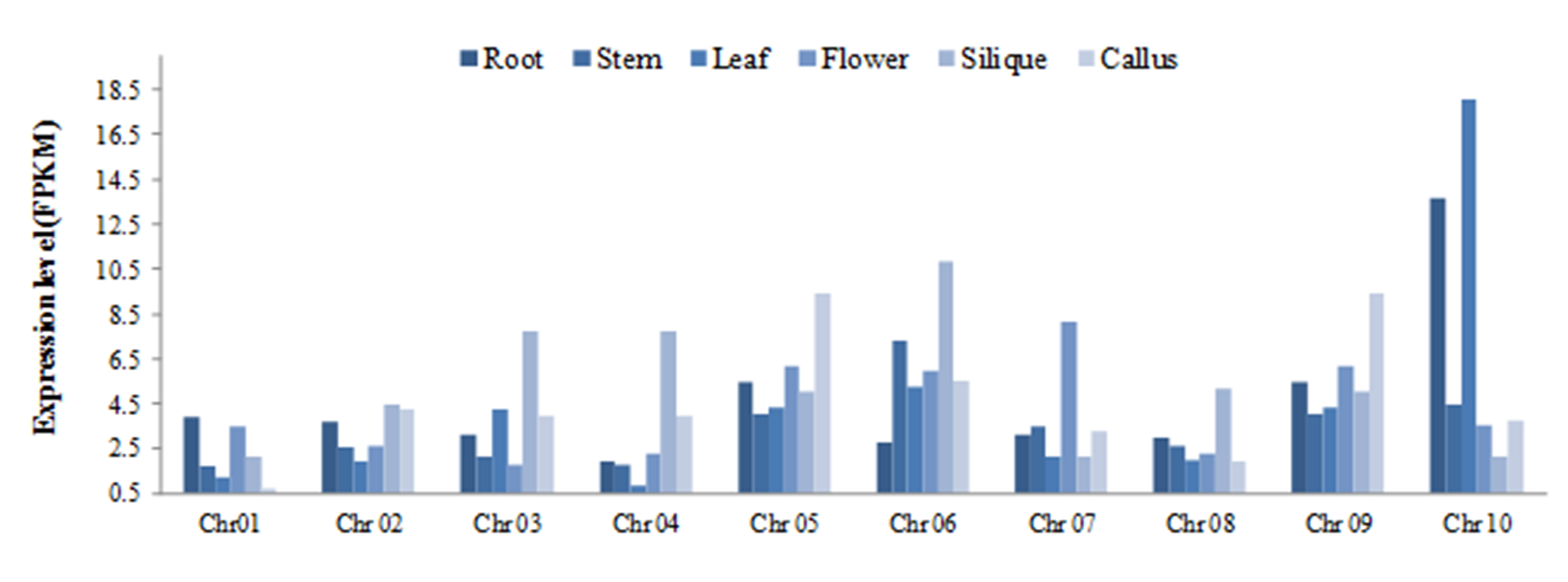

Supplement: S11 Fig — (TIF) [file pone.0256120.s011.tif]

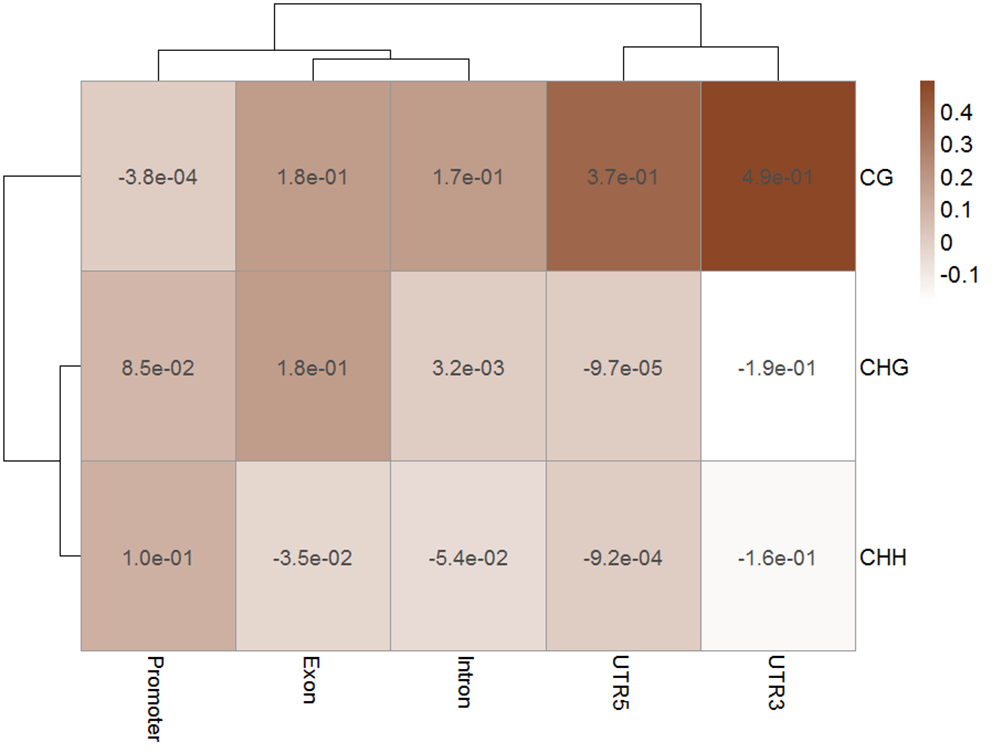

Supplement: S12 Fig — The p values of a paired T-test between PSGs and random non-PSGs by the heat map at CG (a), CHG (b) and CHH (c) contexts in B. rapa. (TIF) [file pone.0256120.s012.tif]

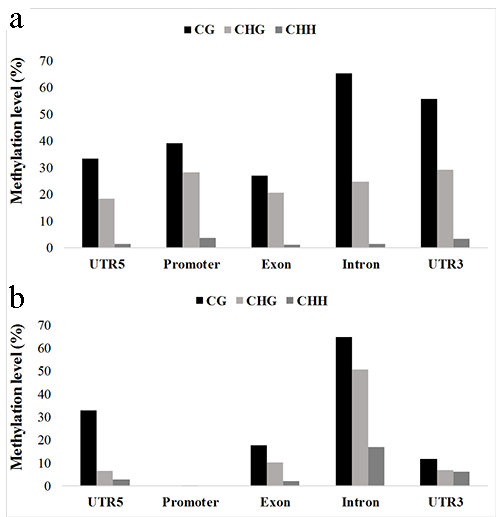

Supplement: S13 Fig — The methylation level of genes inserted by transposable elements (a) or not (b). (TIF) [file pone.0256120.s013.tif]

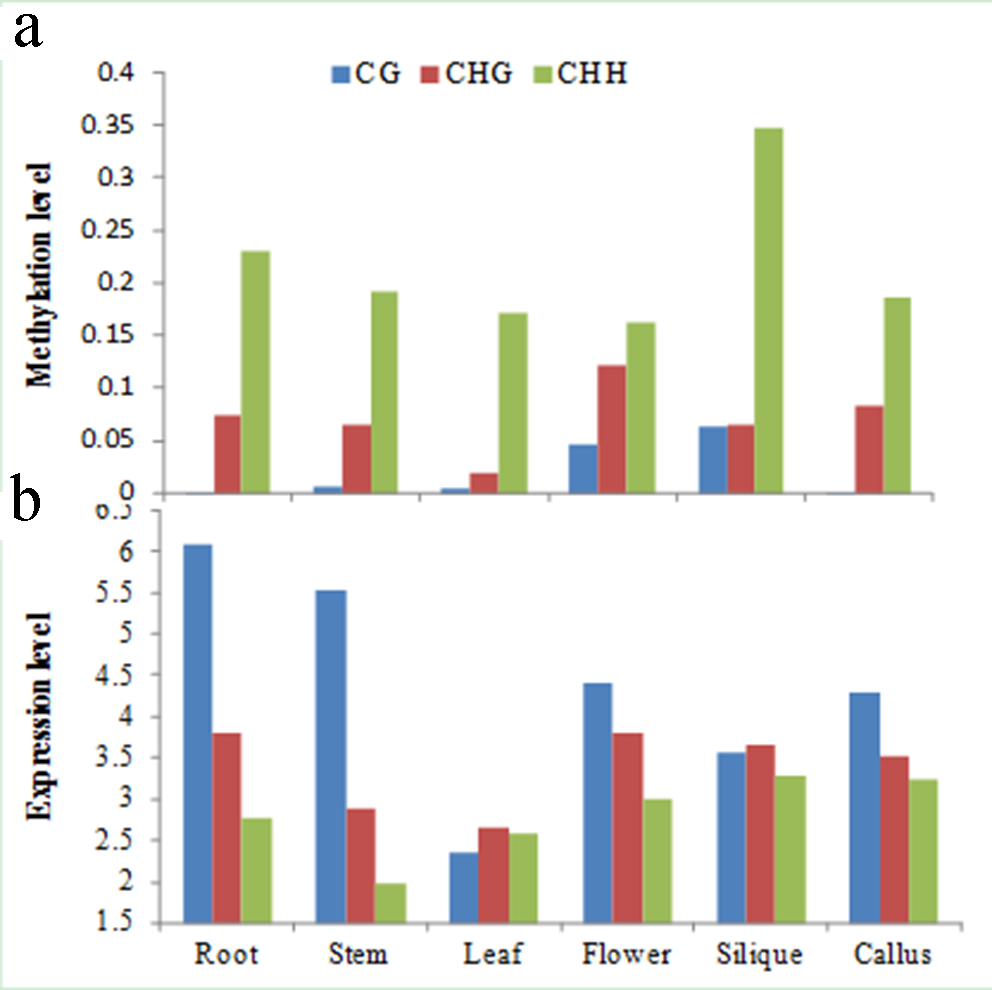

Supplement: S14 Fig — The methylation (a) and expression (b) level of the PSGs in six tissues at three different methylation contexts. (TIF) [file pone.0256120.s014.tif]

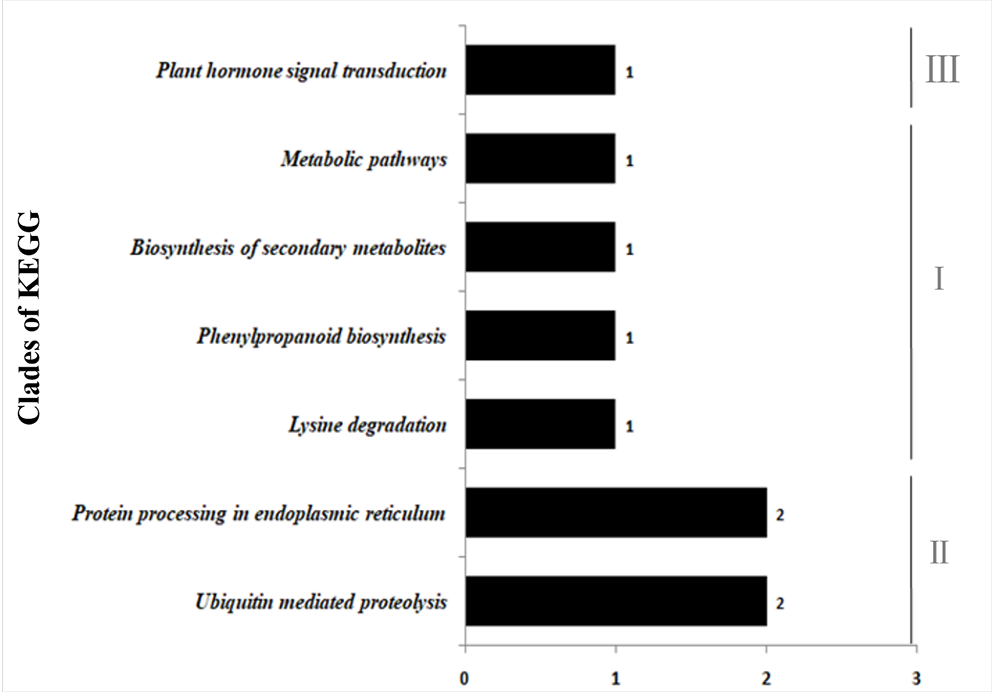

Supplement: S15 Fig — (TIF) [file pone.0256120.s015.tif]

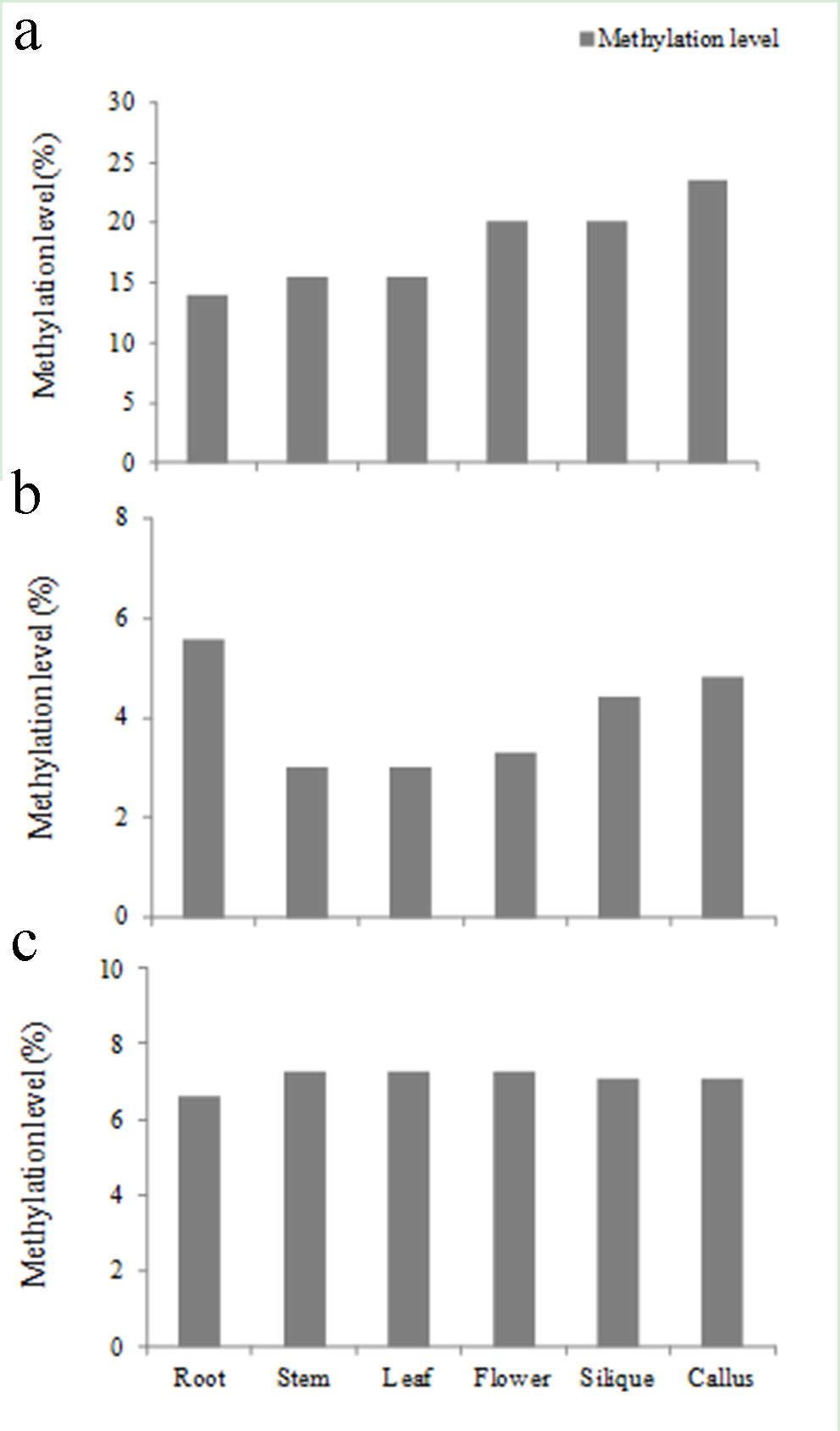

Supplement: S16 Fig — Comparisons of DNA methylation level of the PSGs with constitutive expression in six B. rapa tissues (roots, stems, leaves, flowers, siliques and callus) at CG (a), CHG (b) and CHH (c) contexts. (TIF) [file pone.0256120.s016.tif]
